# In Vitro Oral Cavity Permeability Assessment to Enable Simulation of Drug Absorption

**DOI:** 10.3390/pharmaceutics17070924

**Published:** 2025-07-17

**Authors:** Pankaj Dwivedi, Priyata Kalra, Haiying Zhou, Khondoker Alam, Eleftheria Tsakalozou, Manar Al-Ghabeish, Megan Kelchen, Giovanni M. Pauletti

**Affiliations:** 1 Department of Pharmaceutical and Administrative Sciences, University of Health Sciences and Pharmacy in Saint Louis, St. Louis, MO 63110, USA; 2 Simulations Plus, Inc., Research Triangle Park, NC 27709, USA; 3 Office of Research and Standards, Office of Generic Drugs, Center for Drug Evaluation and Research, U.S. Food and Drug Administration, Silver Spring, MD 20993, USA

**Keywords:** Oral cavity drug delivery, sublingual, buccal, generic drug development

## Abstract

**Background/Objectives:** The oral cavity represents a convenient route of administration for drugs that exhibit significant hepatic first-pass extraction. In this study, the mucosal permeation properties of selected active pharmaceutical ingredients (APIs) incorporated into oral cavity drug products that are approved by the U.S. Food and Drug Administration were quantified using the human-derived sublingual HO-1-u-1 and buccal EpiOral™ in vitro tissue models. **Methods**: Epithelial barrier properties were monitored using propranolol and Lucifer Yellow as prototypic transcellular and paracellular markers. APIs were dissolved in artificial saliva, pH 6.7, and transepithelial flux from the apical to the basolateral compartment was quantified using HPLC. **Results**: Apparent permeability coefficients (Papp) calculated for these APIs in the sublingual HO-1-u-1 tissue model varied from Papp = 2.72 ± 0.06 × 10^−5^ cm/s for asenapine to Papp = 6.21 ± 2.60 × 10^−5^ cm/s for naloxone. In contrast, the buccal EpiOral™ tissue model demonstrated greater discrimination power in terms of permeation properties for the same APIs, with values ranging from Papp = 3.31 ± 0.83 × 10^−7^ cm/s for acyclovir to Papp = 2.56 ± 0.68 × 10^−5^ cm/s for sufentanil. The tissue-associated dose fraction recovered at the end of the transport experiment was significantly increased in the buccal EpiOral™ tissue model, reaching up to 8.5% for sufentanil. **Conclusions**: Experimental permeation data collected for selected APIs in FDA-approved oral cavity products will serve as a training set to aid the development of predictive computational models for improving algorithms that describe drug absorption from the oral cavity. Following a robust in vitro–in vivo correlation analysis, it is expected that such innovative in silico modeling strategies will the accelerate development of generic oral cavity products by facilitating the utility of model-integrated evidence to support decision making in generic drug development and regulatory approval.

## 1. Introduction

Oral cavity drug delivery, where drug absorption occurs immediately after dosage form administration across the mucosa lining the oral cavity, has gained significant interest as an alternative strategy to the conventional oral route of administration, where drug absorption predominantly occurs in the small intestine after transfer through the acidic stomach environment. Therefore, the oral cavity route offers advantages for drug administration in patients unable to swallow. The pharmacokinetic and pharmacodynamic benefits of this non-invasive route of administration include rapid drug absorption into systemic circulation, thereby minimizing first-pass metabolism and/or reducing adverse events (e.g., apomorphine and asenapine) [1,2,3]. Conversely, controlled drug release from oral cavity drug products can effectively facilitate prolonged localized pharmacological effects (e.g., acyclovir buccal tablets in the management of herpes labialis) [4]. The oral cavity route may also be advantageous for the administration of acid-sensitive drugs that are poorly absorbed from the gastrointestinal tract (e.g., peptides) [5].

Histologically, the oral mucosa comprises both keratinized and non-keratinized regions. As non-keratinized epithelia represent a thinner anatomical barrier (i.e., sublingual epithelium 130~190 µm and buccal epithelium 500–800 µm), these oral cavity regions are considered more permeable and are particularly suited for systemic drug delivery. In addition, the sublingual and buccal mucosa are highly vascularized, which facilitates rapid disappearance of absorbed drug from the epithelial barrier and, hence, maintenance of an effective concentration gradient that supports favorable absorption kinetics [6]. Oral cavity drug products designed for either localized or systemic effects encompass a diverse array of formulations ranging from lozenges, buccal/sublingual tablets/films, and sprays to medicated gums. Unless specifically designed as controlled drug delivery systems, the residence time of oral cavity drug products is generally short (<5 min). This requires formulation optimization for rapid disintegration and dissolution to maximize drug absorption within the oral cavity. Due to the absence of significant expression levels of influx and efflux transporters within the non-keratinized regions of the oral cavity mucosa, it is predicted that passive diffusion represents the predominant transmucosal permeation mechanism for drugs released from oral cavity formulations [7]. Consequently, drug absorption from oral cavity drug products seems critically dependent on mucosal permeability and the formulation design, specifically excipients that modulate drug solubility and/or barrier properties of the oral cavity mucosa.

To date, the U.S. Food and Drug Administration (FDA) publication entitled “Approved Drug Products with Therapeutic Equivalence Evaluations”, which is commonly known as the *Orange Book* [8], identifies 13 oral cavity innovator products that are currently marketed within the U.S. Only eight of those have at least one generic equivalent approved by the FDA. To encourage more generic development, the FDA promotes the use and development of computational methods and in silico modeling as one of the main regulatory science research areas. The successful accomplishment of this objective is expected to predict pharmacokinetic plasma profiles after drug product administration within the oral cavity, which represent the results of a complex interplay between drug permeation properties across the oral mucosa, formulation properties, and dosing regimen. The aim of this study was to quantitatively assess intrinsic mucosal permeation properties of active pharmaceutical ingredients (APIs) incorporated in FDA-approved oral cavity drug products (see Table 1) using human-derived in vitro models of the sublingual and buccal tissue barriers. The experimental data collected will serve as a training set for the development of computational in silico models that can simulate drug absorption from the oral cavity under consideration of selected variables such as membrane thickness and drug lipophilicity. Subsequent integration with physiologically based pharmacokinetic modeling may expand the opportunity for mechanistic in vitro–in vivo correlations (IVIVCs) of oral cavity drug products. The generic drug developers may utilize such mechanistic IVIVCs as part of the model-integrated evidence (MIE) approach to support product development including demonstrating bioequivalence of their prospective generics.

## 2. Materials and Methods

### 2.1. Materials

Dulbecco’s modified Eagle’s medium with L-glutamine (DMEM), Ham’s F12 nutrient mixture with L-glutamine, Hanks’ balanced salt solution (HBSS), trypsin-EDTA (0.25%, 1 mM EDTA), penicillin (10,000 IU/mL)-streptomycin (10,000 µg/mL) solution, fetal bovine serum, and other cell culture consumables were supplied by Fisher Scientific (Pittsburgh, PA, USA). Propranolol hydrochloride and Lucifer Yellow were obtained from Sigma-Aldrich (St. Louis, MO, USA). Acyclovir, apomorphine hydrochloride (HCl), asenapine maleate, buprenorphine HCl, fentanyl citrate, naloxone HCl, sufentanil citrate, and zolpidem tartrate were purchased from Sigma-Aldrich. All other chemicals were of analytical or high-performance liquid chromatography (HPLC) grade.

### 2.2. Methods

#### 2.2.1. Cell Culture

HO-1-u-1 cells, derived from a squamous cell carcinoma of the sublingual mucosa exhibiting hyperdiploidy [15], were obtained from the National Institutes of Biomedical Innovation, Health, and Nutrition JCRB Cell Bank (Osaka, Japan). The cells were routinely maintained at 37 °C in a controlled atmosphere of 90% relative humidity and 5% CO_2_ using DMEM/Ham’s F-12 supplemented with 20% (v/v) of heat-inactivated fetal bovine serum, 100 IU/mL of penicillin, and 100 μg/mL of streptomycin. For permeability experiments, HO-1-u-1 cells were seeded onto collagen-coated polyester membranes (Corning^®^ Transwell^®^ # 3450, 4.67 cm^2^, 0.4 µm pore size, Corning Life Sciences, Durham, NC, USA) at a density of 1.5 × 10^5^ cells/well and cultured at 37 °C for two weeks before they were used for transport experiments [16]. The commercial EpiOral™ tissue model (24-well plate, 0.6 cm^2^, 0.4 µm pore size) was obtained from MatTek Corp., (Ashland, MA, USA) and used within 72 h according to the manufacturer’s protocol to assess in vitro buccal permeability.

#### 2.2.2. Permeability Assay Standardization

To ensure consistent and reproducible assay conditions for quantifying API permeation properties, barrier properties of each batch of the sublingual HO-1-u-1 and buccal EpiOral™ tissue models were monitored using propranolol as a prototypic transcellular solute and Lucifer Yellow as a prototypic paracellular solute. Permeation experiments with these markers were performed as described below using 10 µM propranolol and 100 µM Lucifer Yellow in freshly prepared artificial saliva, pH 6.7, that was comprised of 0.75% (*w*/*v*) methylcellulose, 0.1% (*w*/*v*) albumin, and various electrolytes as described by Amal and colleagues [17], but without inclusion of methyl paraben as a preservative. Propranolol was quantified by reverse-phase HPLC with UV detection at λ = 214 nm as outlined in Table 2, while Lucifer Yellow concentrations were measured fluorometrically at EX = 428 nm/EM = 536 nm using a BioTek Synergy™ HT microplate reader (Agilent Technologies, Santa Clara, CA, USA).

#### 2.2.3. Transepithelial Permeation Assessment

API solutions for transport experiments across the sublingual HO-1-u-1 and buccal EpiOral™ tissue models were prepared by combining the API amount equivalent to a single dose of the U.S.-marketed oral cavity drug product (see Table 1) with 5 mL of artificial saliva, pH 6.7, under mild agitation of 50 rpm at 37 °C. These simulated conditions represent a maximum oral cavity hold time of 20 min at an unstimulated saliva flow rate of 0.25 mL/min [25]. Subsequently, the mixture was filtered using a polyvinylidene fluoride (PVDF) syringe filter with 0.45 µm pore size to remove undissolved API. Permeation experiments were initiated by adding this filtrate to the apical donor compartment (i.e., 1.5 mL for HO-1-u-1 and 0.4 mL for EpiOral™ experiments, respectively) and prewarmed HBSS, pH 7.4, to the basolateral receiver compartment (i.e., 2.6 mL for HO-1-u-1 and 0.9 mL for EpiOral™ experiments, respectively). Following protocols previously published by our laboratory [26], a 200 µL aliquot was removed from the basolateral receiver compartment at various time intervals up to 120 min and replaced with fresh, prewarmed HBSS. This timeframe was selected to adequately describe the transepiethelial steady-state flux for each API even in the presence of a lag phase due to limited permeation properties. To quantify the API concentration in the donor compartment at the beginning and end of the transport experiment, a 20 µL aliquot was removed without replacement. Samples were rapidly frozen using a dry ice/ethanol mixture and stored at −20 °C until quantitative HPLC analysis. Permeation studies were performed at 37 °C in a shaking water bath set at 50 rpm. Apparent permeability coefficients (Papp) for each solute were calculated according to Equation (1).

(1) $$Papp=\frac{dQ}{dt}\times \frac{1}{A\times Co}$$

where *dQ*/*dt* = the linear mass appearance rate of the solute in the receiver compartment, *C_0_* = initial solute concentration in the donor compartment, and *A* = surface area available for permeation.

To determine the tissue-associated dose fraction at the end of the permeation experiment and perform mass balance calculations, the epithelial tissue barrier was washed three times with ice-cold HBSS after conclusion of the 120 min transport experiment. Subsequently, the tissue was carefully removed from the filter support using a rubber scraper and diluted with HBSS that contained 20% (*v*/*v*) of acetonitrile. Cell suspensions were sonicated for 10 min on ice, followed by 5 min centrifugation at 4000× *g*. The clear supernatant was collected and processed for quantitative HPLC analysis.

#### 2.2.4. Computational Permeation Prediction

To estimate epithelial permeation of the APIs via passive diffusion using an established in silico prediction tool, we selected the Madin–Darby Canine Kidney low-efflux (MDCK-LE) model Papp predictions in ADMET Predictor^®^ v12 (Simulations Plus, Inc., Research Triangle Park, Durham, NC, USA). MDCK-LE cells represent an MDCK subclone with low efflux transporter activity. They have emerged as a valuable in vitro model to assess epithelial permeation that is primarily driven by passive diffusion without interference by active efflux transporter mechanisms such as P-glycoprotein [27,28]. These predictions were compared with experimentally determined transmucosal permeation data for the APIs across the sublingual HO-1-u-1 and buccal EpiOral™ tissue models with the aim to evaluate the accuracy and applicability of existing computational algorithms for predicting oral mucosal permeability considering the morphological differences between these systems.

#### 2.2.5. HPLC Analysis

Propranolol and oral cavity APIs were quantified using a Shimadzu LC-2050C HPLC system equipped with a quaternary pump, autosampler, and built-in photo diode array detector (Shimadzu Scientific Instruments, Columbia, MA, USA). Isocratic separations were consistently performed at 30 °C using a flow rate of 1 mL/min. Analytical conditions for each solute are delineated in Table 2.

### 2.3. Statistical Analysis

Experiments were carried out at least in triplicate, and results are reported as mean ± standard deviation (S.D.). Statistically significant differences between groups (*p* < 0.05) were evaluated using unpaired Student’s *t*-test or one-way analysis of variance (ANOVA) where appropriate (Prism 10.4, GraphPad, San Diego, CA, USA).

## 3. Results and Discussion

### 3.1. Human-Derived In Vitro Oral Cavity Tissue Models

**Figure 1:**
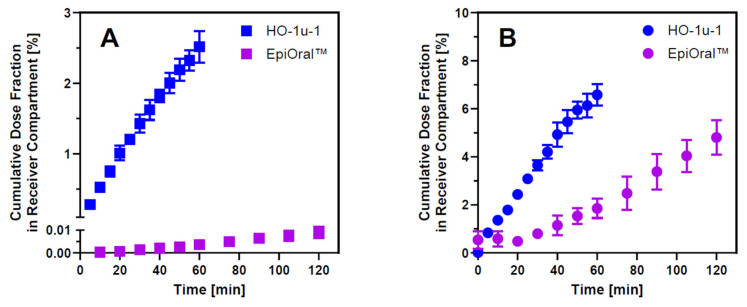
Barrier properties of in vitro oral cavity tissue models. Panel (**A**) = transepithelial flux of the paracellular marker Lucifer Yellow across the sublingual HO-1-u-1 and the buccal EpiOral™ tissue models when administered as a solution in artificial saliva, pH 6.7, to the apical donor compartment. Panel (**B**) = transepithelial flux profiles of the transcellular marker propranolol across both in vitro oral cavity tissue models. Data are shown as mean ± S.D. (*n* = 3).

Successful development of a robust in silico model that can adequately predict the fraction absorbed from the oral cavity in humans requires quantitative mucosal drug permeation data collected systematically under physiologically relevant conditions. Al-though animal models such as the rabbit and pig are still used during drug development, significant cost, ethical concerns, and reliability challenges associated with these in vivo models resulted in the development of viable in vitro alternatives to perform comparative drug absorption studies from the oral cavity [3]. Due to regionally distinct barrier properties of the buccal and sublingual area, transmucosal transfer of drug molecules solubilized in saliva into the submucosal blood supply must be assessed using a combination of at least two different in vitro models representing the buccal and sublingual anatomical regions within the oral cavity. To establish a consistent data set of experimentally determined drug permeation properties for enabling in silico simulation of drug absorption from the oral cavity, the human-derived HO-1-u-1 and EpiOral™ tissue models were selected. Both in vitro models were demonstrated to exhibit histological features that are comparable to the human sublingual and buccal mucosa in vivo [14,29]. Furthermore, these systems are produced under controlled laboratory conditions using conventional cell culture procedures, which increases accessibility and is expected to reduce batch-to-batch as well as within batch variability when compared to animal-derived tissue preparations. Permeability assay conditions were standardized to closely mimic in vivo conditions of oral cavity drug absorption in humans and monitored by quantifying the transepithelial flux of propranolol as a high-permeability marker and Lucifer Yellow as a low-permeability marker across the HO-1-u-1 and EpiOral™ tissue models. The results summarized in Figure 1 underline that mucosal permeability of the sublingual HO-1-u-1 tissue model is significantly higher than the buccal EpiOral™ tissue model for the same marker. On average, the flux of Lucifer Yellow across the sublingual HO-1-u-1 tissue model was almost 400-fold higher when compared to the buccal EpiOral™ tissue model (Papp = 3.57 × 10^−5^ cm/s vs. 9.19 × 10^−8^ cm/s, respectively). In contrast, there was only a 2-fold increase in transepithelial propranolol flux across the sublingual HO-1-u-1 tissue model when compared to the buccal EpiOral™ tissue model (Papp = 3.33 × 10^−5^ cm/s vs. 1.66 × 10^−5^ cm/s, respectively). To decrease batch-to-batch variability, acceptance criteria were defined for Papp values of propranolol and Lucifer Yellow prior to the in vitro assessment of oral cavity permeability for selected APIs using the sublingual HO-1-u-1 and buccal EpiOral™ tissue models.

It is generally expected that in vitro permeability models established on collagen-coated filter support using cell lines such as Caco-2 and MDCK cells form monolayers where paracellular barrier properties are primarily defined by the tight junctions between adjacent cells [30]. The HO-1-u-1 tissue model, however, comprises two to three cell layers that lack tight junctions [14], resulting in a loose intercellular structure where desmosomes and filaments facilitate connections between the squamous epithelial cells, which is consistent with the normal sublingual mucosa in vivo [31]. In comparison to the multilayered architecture of the buccal EpiOral™ tissue model, these histological differences can explain the profound difference in paracellular permeation rate of small hydrophilic molecules such as Lucifer Yellow across this in vitro model of the sublingual mucosa. Propranolol, in contrast, represents a small lipophilic solute that is predicted to permeate the mucosal barrier predominantly by passive diffusion via the transcellular pathway and is frequently used as a high-permeability marker in the method suitability assessment of experimental in vitro models for drug permeability classification [32]. Transmucosal permeation of propranolol is predominantly restricted by the cell membrane composition and the thickness of the tissue barrier. When compared to the EpiOral™ tissue model, the 2-fold increase in propranolol flux across the sublingual HO-1-u-1 tissue model implies that buccal permeability for this transcellular marker seems primarily dependent on the thickness of the EpiOral™ tissue model, which comprises 8–12 cell layers [29].

### 3.2. Permeation Properties of APIs Across In Vitro Oral Cavity Tissue Models

**Figure 2:**
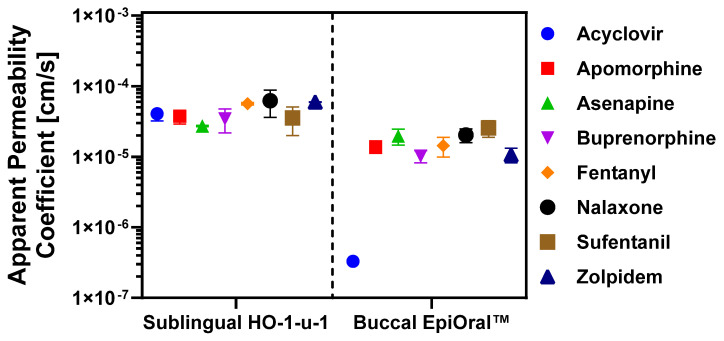
Drug permeation properties across in vitro oral cavity tissue models. Transepithelial API flux was quantified across the sublingual HO-1-u-1 and the buccal EpiOral™ tissue models, respectively. Apical donor solutions were prepared in artificial saliva, pH 6.7, and drug concentrations in basolateral receiver compartment were determined by HPLC. Apparent permeability coefficients (Papp) were calculated from the linear portion of the time-dependent API flux profile. Data are shown as mean ± S.D. (*n* = 6).

Selection of APIs for this study was driven by the desire to develop a reliable and versatile in silico oral cavity absorption model, built using in vitro permeation data measured across the sublingual HO-1-u-1 and the buccal EpiOral™ tissue models and clinical pharmacokinetic data to predict the in vivo performance of oral cavity products applied locally with the intention to act locally or systemically. As the availability of clinical pharmacokinetic profiles after administration of oral cavity drug products in human subjects is limited, the API training set shown in Table 1 was primarily established using the list of FDA-approved oral cavity products. Figure 2 summarizes experimentally determined permeation properties of the selected APIs across the two in vitro oral cavity tissue models. The Papp values calculated from the linear transmucosal API flux across the sublingual HO-1-u-1 tissue model varied from Papp = 2.72 ± 0.06 × 10^−5^ cm/s for asenapine to Papp = 6.21 ± 2.60 × 10^−5^ cm/s for naloxone. These values are in the same order of magnitude as recently published ex vivo Papp values determined for small molecular weight solutes ranging in lipophilicity from logP = −0.1 to 3.9 that were measured across excised sublingual mucosa isolated from the floor of the mouth region in New Zealand White rabbits [33]. The consistent outcome reported for a diverse solute set using either ex vivo rabbit tissue preparations and the in vitro HO-1-u-1 tissue model underline the longstanding conclusion that the sublingual mucosa represents a “leaky” permeation barrier [31]. Statistical comparison of Papp values calculated for these APIs across the sublingual HO-1-u-1 tissue model using ANOVA only reveals significant differences between naloxone and asenapine, apomorphine, and buprenorphine, respectively. This finding underlines the limited capabilities of the sublingual mucosa to effectively discriminate permeation properties of solutes exhibiting different physicochemical properties. Moreover, similar permeation rates measured for lipophilic solutes such as propranolol and hydrophilic solutes such as Lucifer Yellow suggest that the surface area available for transcellular and paracellular diffusion across the sublingual HO-1-u-1 tissue model may be comparable. This is in stark contrast to the 400-fold difference in Papp values observed for these two prototypic marker solutes using the buccal EpiOral™ tissue model, which implies a significantly greater surface area available for transcellular than for paracellular passive diffusion across the buccal mucosal barrier.

Papp values calculated in the buccal EpiOral™ tissue model demonstrate greater discrimination power of permeation properties for the same APIs, ranging from Papp = 3.31 ± 0.83 × 10^−7^ cm/s for acyclovir to Papp = 2.56 ± 0.68 × 10^−5^ cm/s for sufentanil, respectively. The statistical comparison of those Papp values using ANOVA reveals significant differences between acyclovir and all other APIs, whereas buccal permeability for apomorphine was only significantly different from sufentanil. Similarly, Papp value for asenapine was only significantly different from buprenorphine and zolpidem, while buprenorphine was significantly different from naloxone and sufentanil but statistically equivalent to the Papp value of fentanyl and zolpidem, respectively. Consistent with the earlier conclusion that the buccal EpiOral™ tissue model exhibits a surface area that seems more advantageous for passive diffusion of solutes via the transcellular route, the transepithelial flux of lipophilic APIs such as sufentanil (logP = 3.4) was significantly increased when compared to more hydrophilic APIs such as acyclovir (logP = −1.2). It is noted that the correlation between calculated Papp values and API lipophilicity does not seem linear as exemplified by buprenorphine (logP = 4.5; Papp = 1.02 ± 0.21 × 10^−5^ cm/s) and fentanyl (logP = 4.1; Papp = 1.43 ± 0.45 × 10^−5^ cm/s), which list a smaller Papp value than sufentanil (logP = 3.4; Papp = 2.56 ± 0.68 × 10^−5^ cm/s). Transcellular diffusion across a mucosal barrier requires several sequential partitioning events of the solute from an aqueous into a lipophilic environment (e.g., extracellular—membrane bilayer partitioning) and vice versa before reaching the submucosal space. Wanasathop and colleagues delineated parallel lipoidal and polar transport pathways for a diverse set of solutes across porcine buccal mucosa [34]. The analysis by these investigators revealed that the pH-dependent aqueous distribution coefficient rather than the logP value of a solute is a more robust predictor of buccal mucosa permeability. In addition, the correlation improved with incorporation of molecular weight as an additional independent variable. A similar approach can be explored with confidence after completion of additional in vitro permeation studies across the EpiOral™ tissue model using a significantly larger set of APIs that exhibit diverse physiochemical properties. For highly lipophilic solutes such as buprenorphine, it is also conceivable that the transepithelial flux is reduced due to nonspecific binding to lipophilic cellular structures, including membrane bilayers. Conversely, permeation of highly polar solutes such as the amphoteric acyclovir across the buccal mucosa seems limited to the aqueous paracellular pathway that is predicted to offer a significantly smaller surface area for diffusion than the transcellular pathway. This may explain the substantially reduced transepithelial flux of acyclovir that was measured across the buccal EpiOral™ tissue model when compared to the sublingual HO-1-u-1 tissue model. It is noted that buccal permeability for the prototypic paracellular marker Lucifer Yellow, which exhibits similar lipophilic properties as acyclovir (logP = −1.34 [35] vs. logP = −1.37 reported for acyclovir in Table 1), was reduced by more than two orders of magnitude when compared to the sublingual HO-1-u-1 tissue model. Interestingly, acyclovir is the only FDA-approved API included in this study that is formulated as a mucoadhesive sustained-release tablet designed for local pharmacological effect in the oral cavity. The design of this dosage form provides for prolonged contact of a drug reservoir with the buccal mucosa, thereby reducing drug loss in saliva, while maintaining a consistent tissue level of the drug for therapeutic intervention.

The predicted Papp values and corresponding compound rankings generated using the low-efflux Madin–Darby Canine Kidney cell (MDCK-LE) in silico model in ADMET Predictor v12, were analyzed in relation to experimentally determined transmucosal permeation data using the sublingual HO-1-u-1 and buccal EpiOral™ tissue models. The rationale for employing the MDCK-LE computational model predictions stemmed from its utility in estimating passive permeation across epithelial barriers characterized by minimal expression of active influx and efflux transport mechanisms for ranking compounds. It is noted that the rank order shown in Table 3 for experimentally determined Papp values is based on the mean value only to match the same approach as used for the predicted Papp values. Future work will focus on a more robust comparison between observed and in silico predicted permeation properties that includes a parameter sensitivity analysis, which is representative of the inherent variability associated with the experimental procedure. Despite the limitations of this initial comparison, review of this compilation in Table 3 reveals reasonably close agreement between MDCK-LE predictions and experimental Papp values using the buccal EpiOral™ tissue model, except for acyclovir. However, the ranking of acyclovir as the lowest permeability compound is in concordance for buccal measurements and in silico MDCK-LE prediction. MDCK-LE cells represent a subpopulation of the MDCKII wild type cell line exhibiting significantly decreased expression of endogenous membrane efflux transporter activity such as P-glycoprotein. It is noted that MDCK-LE cells maintain distinct characteristics of the wild type cells such as low expression of metabolizing enzymes and transporters, morphologic homogeneity, high monolayer integrity, and tight paracellular junctions [27]. The latter property is hypothesized to contribute to the deviation between the predicted Papp value and the experimental Papp value determined using the sublingual HO-1-u-1 tissue model, which lacks tight junctions [14]. For some of the lipophilic APIs such as fentanyl and zolpidem, computational permeation predictions using the MDCK-LE cell model suggested a significantly greater transepithelial API flux than was measured experimentally using the buccal EpiOral™ tissue model. These discrepancies may be the result of morphological differences as MDCK-LE cells retain their ability to form microvilli on the apical membrane, which increases the surface area available for transcellular passive diffusion for lipophilic solutes. In contrast, the buccal EpiOral™ tissue model histologically and phenotypically resembles the native buccal mucosa without microvilli [29]. Furthermore, the fundamental differences in architecture-comparing a monolayer (MDCK-LE) to the multilayered structures of the HO-1-u-1 and EpiOral™ models-along with the increased tissue thickness inherent to the latter, create distinct diffusion pathways and barrier characteristics not fully captured by the simpler monolayer system. Additionally, pH differences between experimental tissue models (i.e., pH 6.7) and computational predictions (i.e., pH 7.4) can alter ionization of selected API that affects permeability outcomes.

In conclusion, the comparison between predicted permeability using the MDCK-LE cell-based ADMET Predictor Papp model and experimental data from the HO-1-u-1 and EpiOral™ systems demonstrated varying degrees of agreement, highlighting the inherent complexity of accurately modeling drug permeability across different in vitro representations of the oral mucosa. These findings underscore the need for cautious interpretation of computational predictions and highlight the importance of cross validating such results using multiple experimental systems. For instance, combining the sensitivity of the buccal EpiOral™ tissue model with the physiological relevance of the sublingual HO-1-u-1 tissue model could refine oral cavity permeability assessments. While computational tools like ADMET Predictor offer valuable insights for screening and ranking hypothesis generation, they remain supplementary to experimental models, particularly for drugs with complex absorption profiles. Addressing morphological mismatches, pH variations, and tissue-specific properties will be critical for improving predictive accuracy of computational prediction tools and advancing the development of oral cavity drug products.

### 3.3. Tissue Binding

**Figure 3:**
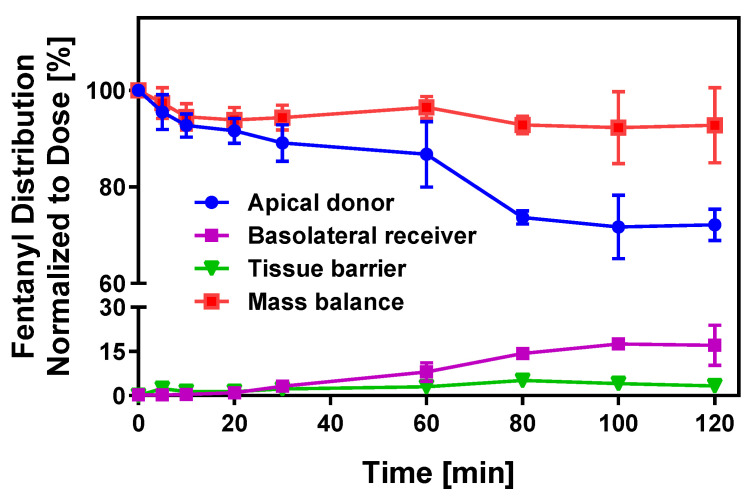
Transport kinetics of fentanyl across the buccal EpiOral™ tissue model. Time-dependent drug distribution in apical donor, basolateral receiver, and epithelial tissue compartments during the in vitro transport experiment of fentanyl across the buccal EpiOral™ tissue model was quantified by HPLC and normalized to the total API dose administered to the donor compartment at t = 0 min. Data are shown as mean ± S.D. (*n* = 6).

Drug transfer from the oral cavity into submucosal blood vessels is not only dependent on tissue permeability and dissolved API concentration at the site of absorption but also influenced by a bidirectional partitioning process between saliva and mucosal tissue. It is predicted that mucosal tissue rapidly equilibrates with the API solution in the oral cavity. Despite rapid drug disappearance after oral cavity administration, epithelial tissue may retain a substantial dose fraction due to non-specific binding, which can significantly delay drug appearance in the systemic circulation [36,37]. To delineate the various kinetic processes driving transmucosal absorption across the multilayered, stratified buccal tissue, time-dependent drug distribution within distinct compartments of the buccal EpiOral™ tissue model (i.e., apical donor and basolateral receiver compartments as well as the epithelial tissue barrier) was quantitatively monitored using fentanyl (Figure 3).

The kinetic profile for this API in the donor compartment indicates rapid disappearance of ~10% of the dose administered within 20 min without substantial drug accumulation in the receiver compartment (<0.5%). Within five minutes, however, fentanyl content in the epithelial tissue barrier increases to ~2% and remains stable at 3–4% until the end of the in vitro transport experiment. After a 30 min lag time, the API amount transported into the basolateral receiver compartment increases, reaching a maximum value of 17% after 120 min. In parallel, the drug amount in the donor compartment gradually decreases, which is consistent with the directional API transport across the buccal EpiOral™ tissue model. Mass balance calculations demonstrate a high recovery of >95% throughout the experiment. It is hypothesized that the unaccounted fraction of this lipophilic API is the consequence of nonspecific binding to the polystyrene plastic support used in this experimental design. The results from these experiments strongly support the conclusions from earlier studies that suggested rapid equilibration between the oral cavity drug concentration and epithelial barrier.

**Figure 4:**
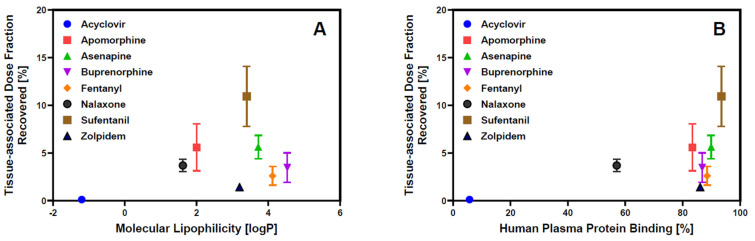
Epithelial API retention in the in vitro buccal EpiOral™ tissue model. Panel (**A**) = Comparison of tissue-associated drug content at the end of the 120 min in vitro transport experiment across the buccal EpiOral™ tissue model with API’s molecular lipophilicity reported in Table 1. Panel (**B**) = comparison of tissue-associated drug content at the end of the 120 min in vitro transport experiment across the buccal EpiOral™ tissue model with API’s human plasma protein binding reported in Table 1. Data are shown as mean ± S.D. (*n* = 6).

The dose fraction associated with the epithelial tissue barrier after API release from the oral cavity dosage form can serve as a reservoir, enabling continuous drug absorption after removal of the oral cavity drug by swallowing. In parallel, the API can partition back into saliva, thereby reducing the concentration gradient that drives absorption into the systemic circulation [36,37]. Experimental assessment of the tissue-associated dose fraction of the APIs recovered at the end of the transport experiment across the buccal EpiOral™ tissue model varied between 0.1% for acyclovir and 8.5% for sufentanil. Although the mechanisms of nonspecific tissue binding are complex, it is hypothesized that physicochemical API properties such as lipophilicity are critical for drug retention within the mucosal barrier. Figure 4A visualizes the correlation between the tissue-associated dose fraction quantified after conclusion of the transport experiment using the buccal EpiOral™ tissue model and the logP value of each API as a measure of molecular lipophilicity. The low level of retention measured for acyclovir suggests limited affinity of this polar API for cellular components of the EpiOral™ tissue barrier. Assuming predominant permeation of acyclovir via paracellular diffusion, these results may also imply that the passage of this API across the cell barrier is limited due to the presence of polar, but non-ionic, intermolecular forces such as hydrogen bonding. In contrast, lipophilic APIs such as sufentanil demonstrate a significantly greater affinity for cellular components of the EpiOral™ tissue barrier, suggesting that cumulative hydrophobic interactions during transcellular diffusion facilitate substantial drug retention within the epithelial barrier.

Nevertheless, molecular lipophilicity as represented by the APIs’ logP values does not appear to adequately explain the ranking order of the experimentally recovered tissue-associated dose fraction at the end of the 120 min in vitro transport experiment across the buccal EpiOral™ tissue model. Figure 4B depicts the correlation between tissue-associated drug content at the end of the 120 min in vitro transport experiment across the buccal EpiOral™ tissue model and human plasma protein binding reported for the various APIs in Table 1. In this representation, the ranking order of APIs seems to allow for more effective delineation between APIs exhibiting limited affinity for this in vitro buccal tissue model such as acyclovir and other APIs, including apomorphine and sufentanil, that demonstrate significantly increased tissue retention. It is hypothesized that consideration of API- and protein-specific ionization that contribute to plasma protein binding in addition to molecular lipophilicity are likely responsible for this improved correlation in ranking order.

## 4. Conclusions

This study demonstrates the utility of the sublingual HO-1-u-1 and buccal EpiOral™ tissue models in quantifying intrinsic in vitro drug permeation properties across distinct anatomical regions of the oral cavity. Both human-derived cell culture models are produced under controlled laboratory conditions, which enables reproducible permeability assessment using standardized protocols that closely mimic the in vivo conditions of oral cavity drug absorption in humans. The results from these in vitro permeation experiments indicate that the multilayered and stratified buccal mucosa exhibits greater discrimination power in permeability for APIs with different physicochemical properties than the sublingual mucosa, which is considered a “leaky” epithelial barrier due to the absence of tight junctions. Drug concentrations between the oral cavity and epithelial tissue barriers appear to rapidly equilibrate, while the tissue-associated dose fraction seems to correlate with physicochemical API properties, including molecular lipophilicity and ionization, that also contribute to plasma protein binding. The experimental data collected for this series of APIs that are incorporated in FDA-approved oral cavity products will serve as a training set to aid development of predictive computational models for improving algorithms that describe drug absorption from the oral cavity. Subsequent integration with physiologically based pharmacokinetic modeling may expand the opportunity for mechanistic in vitro–in vivo correlations to support generic developers of oral cavity drug products with model-integrated evidence (MIE) that can mitigate the risk of failure mode for bioequivalence and enable optimization of bioequivalence study design. To increase robustness of such MIE approaches for generic drug development, effects of different excipients on dissolution properties of final dosage forms as well as excipient-induced modulations of mucosal barrier properties restricting API permeation should be explored in the future.

## Figures and Tables

**Table 1:** APIs in FDA-approved oral cavity products selected for in vitro sublingual and buccal permeability assessments.

Active Pharmaceutical Ingredient	Molecular Weight [Da]	pKa ^1)^	Lipophilicity [logP] ^1)^	Human Plasma Protein Binding ^1)^	U.S.-Marketed Single Dose ^2)^	Oral Bioavailability	Generic Equivalent
Acyclovir	225.3	2.53; 1.19	−1.37	5.7%	50 mg (buccal)	15–30% [9]	No
Apomorphine hydrochloride	303.8	7.64	2.81	83.4%	30 mg (sublingual)	1.7% [2]	No
Asenapine maleate	401.8	8.57	4.20	89.9%	10 mg (sublingual)	2% [3]	Yes
Buprenorphine hydrochloride	504.1	8.21	4.92	86.8%	8 mg (sublingual)	>20% [10]	No
Fentanyl citrate	528.6	8.15	3.99	88.5%	800 µg (sublingual)	<10% [11]	No
Naloxone hydrochloride	363.8	7.31	1.25	57.0%	2 mg (buccal/sublingual)	<2% [12]	No
Sufentanil citrate	578.7	7.94	3.86	93.5%	30 µg (sublingual)	<10% [13]	No
Zolpidem tartrate	764.9	5.53	2.77	86.1%	10 mg (sublingual)	45–70% [14]	Yes

^1)^ Computational predictions using ADMET Predictor v12. ^2)^ Single dose of oral cavity product selected for this study.

**Table 2:** Chromatographic separation conditions.

Analyte	Stationary Phase	Mobile Phase	Retention Time	Detection Wavelength	Ref.
Acyclovir	C_18_ (250 mm × 4.6 mm, 5 µm particle size)	10 mM phosphate buffer, pH 4.5 supplemented with 0.05% (w/v) of 1-decane sulfonic acid/ACN (95:5)	4.1 min	254 nm	[18]
Apomorphine HCl	C_18_ (250 mm × 4.6 mm, 5 µm particle size)	50 mM o-phosphoric acid, pH 3.1/ACN (80:20)	5.8 min	272 nm	[19]
Asenapine maleate	C_18_ (250 mm × 4.6 mm, 5 µm particle size)	25 mM phosphate buffer, pH 3.2/ACN (95:5)	3.7 min	232 nm	[20]
Buprenorphine HCl	C_8_ (150 mm × 4.6 mm, 5 µm particle size)	10 mM phosphate buffer, pH 6.0 supplemented with 0.05% (w/v) of 1-decane sulfonic acid/ACN (35:65)	7.5 min	214 nm	[21]
Fentanyl citrate	C_18_ (250 mm × 4.6 mm, 5 µm particle size)	50 mM phosphate buffer, pH 4.5/ACN (65:35)	4.5 min	230 nm	[22]
Naloxone HCl	C_18_ (250 mm × 4.6 mm, 5 µm particle size)	10 mM phosphate buffer, pH 5.0/ACN (80:20)	4.8 min	283 nm	[21]
Propranolol	C_18_ (250 mm × 4.6 mm, 5 µm particle size)	50 mM phosphate buffer, pH 4.5/ACN (65:35)	6 min	214 nm	[23]
Sufentanil citrate	C_18_ (250 mm × 4.6 mm, 5 µm particle size)	130 mM ammonium acetate buffer, pH 7.2/ACN (40:60)	6.8 min	230 nm	[22]
Zolpidem tartrate	C_18_ (250 mm × 4.6 mm, 5 µm particle size)	20 mM ammonium acetate buffer, pH 8.0/ACN (40:60)	5.2 min	245 nm	[24]

**Table 3:** Comparison of experimental permeation properties and permeability ranking of APIs across in vitro models of the oral cavity with computational permeation predictions using the MDCK-LE Model.

Active Pharmaceutical Ingredient	Sublingual Papp/Ranking ^1)^ [×10^5^ cm/s]	Buccal Papp/Ranking ^2)^ [×10^5^ cm/s]	Papp MDCK-LE Predicted/Ranking ^3)^ [×10^5^ cm/s]
Acyclovir	4.07 ± 0.87 (#4)	0.03 ± 0.01 (#8)	0.15 (#8)
Apomorphine HCl	3.73 ± 0.82 (#5)	1.38 ± 0.27 (#5)	1.90 (#4)
Asenapine maleate	2.72 ± 0.06 (#8)	1.96 ± 0.50 (#3)	1.89 (#3)
Buprenorphine HCl	3.45 ± 1.28 (#7)	1.02 ± 0.21 (#7)	1.25 (#7)
Fentanyl citrate	5.63 ± 0.23 (#3)	1.43 ± 0.45 (#4)	2.32 (#1)
Naloxone HCl	6.21 ± 2.60 (#1)	2.30 ± 0.45 (#2)	1.76 (#6)
Sufentanil citrate	3.54 ± 1.54 (#6)	2.56 ± 0.68 (#1)	1.87 (#5)
Zolpidem tartrate	5.97 ± 0.05 (#2)	1.07 ± 0.25 (#6)	2.25 (#2)

^1)^ Experimentally determined across the sublingual HO-1-u-1 tissue model (*n* = 6). ^2)^ Experimentally determined across the buccal EpiOral™ tissue model (*n* = 6) ^3)^ Computational permeation predictions using the MDCK-LE cell model in ADMET Predictor v12. # = ranking position within the data series.

## Data Availability

The original data presented in the study are openly available in FigShare.
